# Effects of Chikungunya virus immunity on Mayaro virus disease and epidemic potential

**DOI:** 10.1038/s41598-019-56551-3

**Published:** 2019-12-31

**Authors:** Emily M. Webb, Sasha R. Azar, Sherry L. Haller, Rose M. Langsjoen, Candace E. Cuthbert, Anushka T. Ramjag, Huanle Luo, Kenneth Plante, Tian Wang, Graham Simmons, Christine V. F. Carrington, Scott C. Weaver, Shannan L. Rossi, Albert J. Auguste

**Affiliations:** 1 Department of Entomology, Fralin Life Science Institute, Virginia Polytechnic Institute and State University, Blacksburg, VA 24061 USA; 2 Institute for Human Infections and Immunity, and Department of Microbiology and Immunology, University of Texas Medical Branch, Galveston, TX 77555 USA; 3 Institute for Translational Sciences, University of Texas Medical Branch, Galveston, TX 77555 USA; 4 Department of Preclinical Sciences, Faculty of Medical Sciences, The University of the West Indies, St. Augustine, Trinidad and Tobago; 5 World Reference Center for Emerging Viruses and Arboviruses, University of Texas Medical Branch, Galveston, TX 77555 USA; 6 Vitalant Research Institute, 270 Masonic Avenue, San Francisco, CA 94118 USA

**Keywords:** Infectious diseases, Viral infection

## Abstract

Mayaro virus (MAYV) causes an acute febrile illness similar to that produced by chikungunya virus (CHIKV), an evolutionary relative in the Semliki Forest virus complex of alphaviruses. MAYV emergence is typically sporadic, but recent isolations and outbreaks indicate that the virus remains a public health concern. Given the close phylogenetic and antigenic relationship between CHIKV and MAYV, and widespread distribution of CHIKV, we hypothesized that prior CHIKV immunity may affect MAYV pathogenesis and/or influence its emergence potential. We pre-exposed immunocompetent C57BL/6 and immunocompromised A129 or IFNAR mice to wild-type CHIKV, two CHIKV vaccines, or a live-attenuated MAYV vaccine, and challenged with MAYV. We observed strong cross-protection against MAYV for mice pre-exposed to wild-type CHIKV, and moderately but significantly reduced cross-protection from CHIKV-vaccinated animals. Immunity to other alphavirus or flavivirus controls provided no protection against MAYV disease or viremia. Mechanistic studies suggested that neutralizing antibodies alone can mediate this protection, with T-cells having no significant effect on diminishing disease. Finally, human sera obtained from naturally acquired CHIKV infection cross-neutralized MAYV at high titers *in vitro*. Altogether, our data suggest that CHIKV infection can confer cross-protective effects against MAYV, and the resultant reduction in viremia may limit the emergence potential of MAYV.

## Introduction

Mayaro virus (MAYV) is an arthropod-borne virus and a member of the family *Togaviridae*, genus *Alphavirus*. Since its isolation from Trinidadian forest workers in 1954^1^, MAYV has become of increasing concern for the neotropics^2–4^. MAYV is the etiological agent of Mayaro fever (MAYF), a disease often misdiagnosed due to its similar clinical presentation to dengue and chikungunya fevers, as well as many other tropical diseases. The co-circulation of these viruses in many Latin American countries further complicates accurate diagnoses^5–7^. Infection with MAYV typically results in an acute febrile illness, with flu-like signs and symptoms such as a cutaneous rash, headache, myalgia, and debilitating arthralgia that can persist for months or, in some cases, years following infection^8^. MAYV is a 65–70 nm enveloped virus with a ~11.5 kb single-stranded, positive-sense genomic RNA that is packaged within a nucleocapsid. The genome encodes five structural proteins (C, E1-3, and 6K), including capsid and envelope proteins, and four non-structural proteins (nsP1-4), encoding the virus’ replication machinery^9^. Phylogenetic studies have revealed three distinct MAYV lineages: genotype D (widely dispersed), genotype L (more limited distribution detected), and genotype N (newly identified)^10^. Genotypes D and L are considered major lineages whereas genotype N consists of one strain isolated in Peru in 2010^10^. Genotype L has been reported only in Brazil and Haiti, while genotype D contains isolates from Trinidad, Suriname, French Guiana, Peru, Bolivia, Venezuela, and Brazil^11^. Recently, MAYV exposure has been detected serologically or by virus detection in countries ranging as far south as Brazil and as far north as Mexico^12–22^.

MAYV outbreaks are typically relatively small and occur primarily in Brazil and Peru^8,11,22–25^; however, MAYV has demonstrated its potential for large outbreaks such as the ~800 persons affected in Brazil in 1978^22^. Interestingly, a recent case in Haiti reported an eight-year-old boy presenting with fever, abdominal pain, who was co-infected with dengue virus (DENV) and MAYV^13^. The detection of MAYV is unprecedented in Haiti and surprising due to the absence of indigenous nonhuman primates (NHP; key amplification hosts in MAYV’s proposed enzootic cycle) in Haiti. While this cycle has not been fully characterized, MAYV is believed to circulate between canopy-dwelling *Haemagogus* mosquitoes and NHPs, similar to sylvatic yellow fever virus in the neotropics^26^. However, in 2003, Thoisy *et al*. conducted a serological survey for MAYV in wild animals from French Guiana and found seropositive birds, rodents, NHPs, and other small mammals^18^. Furthermore, mosquitoes from the *Aedes* genus (*e.g. Ae. albopictus* and *Ae. aegypti*) can experimentally transmit MAYV^27,28^, and MAYV has been isolated from *Ae. aegypti* and possibly *Culex quinquefasciatus* mosquitoes in the wild^29^. Recent studies show that *Culex quinquefasciatus* are incompetent MAYV vectors but highlight the potential importance of *Anopheline* mosquitoes in transmission^30^. These data, coupled with recent outbreaks, suggest the existence of alternative enzootic vertebrate hosts, undescribed vectors, and/or the beginning of MAYV’s adaptation to an urban or peridomestic epidemic transmission cycle.

Chikungunya virus (CHIKV) is a close alphavirus relative of MAYV and a fellow member of the Semliki Forest complex. CHIKV infection results in a similar disease presentation, characterized by persistent, debilitating arthralgia, and has a comparable sylvatic transmission cycle to that of MAYV, but in sub-Saharan Africa. However, it has a long history of emergence into a peridomestic, human-amplified cycle and of invading Asia, the Indian Ocean Basin^31^, and recently the Americas in 2013^32^. CHIKV is now among the most widely distributed alphaviruses and circulates in all countries with evidence of MAYV transmission^33^.

Given the similarities in disease presentation and potentially similar peridomestic transmission cycles, as well as the close phylogenetic and antigenic relationship between MAYV and CHIKV, we hypothesized that the recent CHIKV spread throughout the Americas and resultant herd immunity in humans, as well as the potential for CHIKV to establish enzootic transmission there, will affect transmission and spread of MAYV in the Americas. Previous studies have been conducted to understand cross-protective immunity among alphaviruses and, specifically, viruses within the Semliki Forest virus complex^34–40^. A live-attenuated CHIKV vaccine candidate (*e.g*. CHIKV/IRES) elicits a strong cross-neutralizing antibody response to o’nyong-nyong virus (ONNV), a close relative of CHIKV, and provides complete protection from ONNV but not Ross river virus disease in mouse models^34^. This study suggests that neutralizing antibodies are the sole mediator of the observed cross-protection. Furthermore, Fox *et al*. screened a panel of anti-CHIKV mouse monoclonal antibodies (mAbs) for cross-neutralizing potential against other alphaviruses (*i.e*. ONNV, MAYV, Semliki Forest virus, and Ross River virus), and demonstrated that a single anti-CHIKV mouse mAb completely protects mice from MAYV disease and mortality^36^.

Given the phylogenetic and serological relationship between CHIKV and MAYV, we investigated the potential effects of CHIKV immunity on MAYV infection and disease using established immunocompetent and immunocompromised murine models^36,41,42^. Additionally, we studied the potential application of leading CHIKV vaccine candidates to control MAYV disease and potential emergence. Our results demonstrate that immunity to CHIKV can significantly reduce murine MAYV disease and that neutralizing antibodies alone can mediate this protection, with T-cells not playing a significant role in protection. Our data also show that naturally acquired CHIKV immunity in humans can neutralize MAYV *in vitro*. These data also suggest a certain threshold of CHIKV immunity is needed to protect against MAYV disease, such that the chimeric or live-attenuated CHIKV vaccines are unlikely to control a MAYV outbreak, while naturally acquired CHIKV immunity (wild-type CHIKV infection) may be adequate to provide protection and reduce viremias sufficiently to impede outbreaks.

## Results

### Wild-type CHIKV protects against MAYV disease in immunocompetent mice

**Figure 1:**
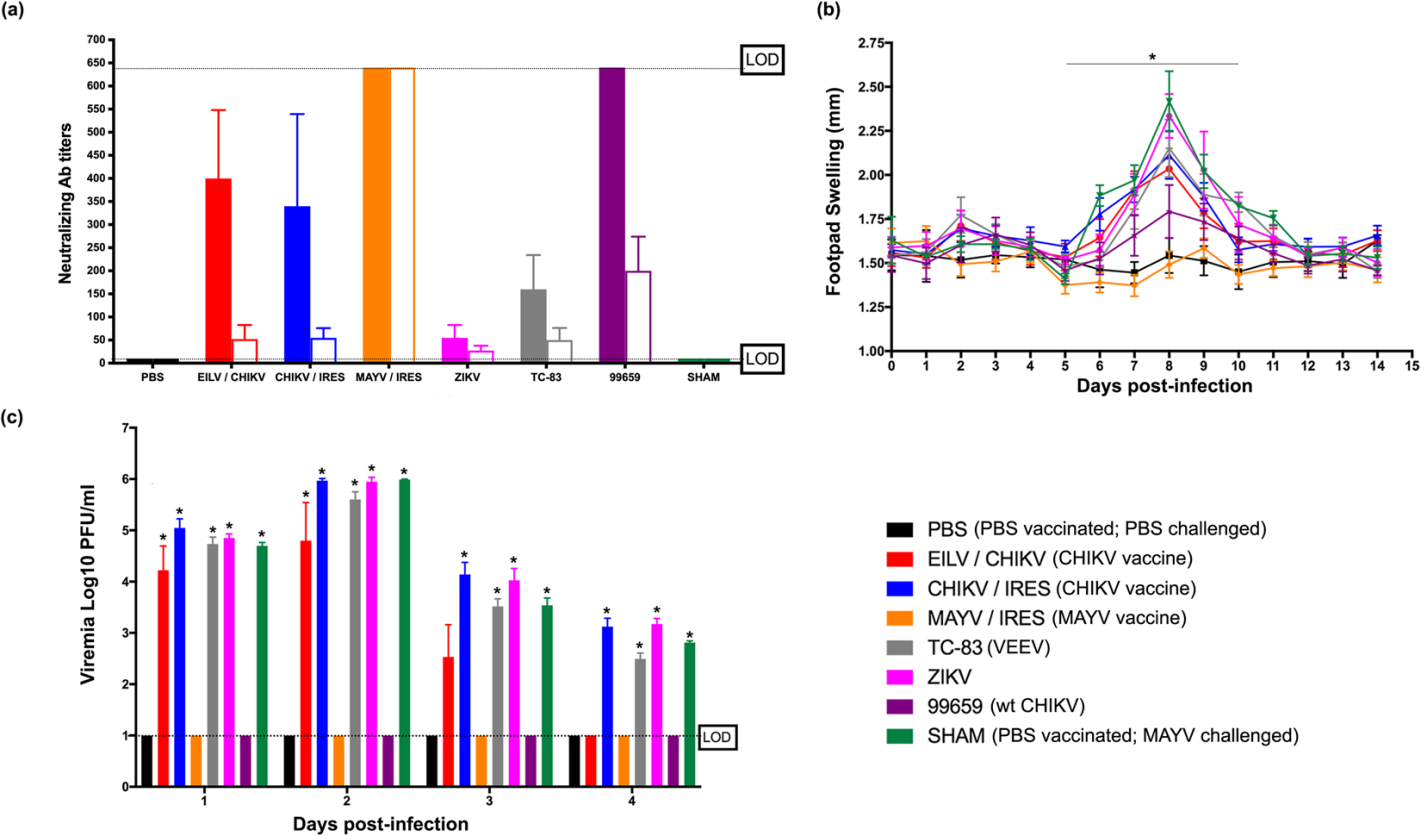
CHIKV immunity provides cross-protection against MAYV infection and disease in an immunocompetent mouse model. (**a**) Virus-specific neutralizing antibody titers (solid bars) and MAYV cross-neutralization antibody titers (empty bars) were determined prior to MAYV challenge. Dashed lines indicate the upper and lower limits of detection (1:640 and 1:20, respectively); (**b**) footpad swelling were determined daily throughout the study; (**c**) Viremia was measured daily for four days post-infection. All plotted values in (**b,c**) are mean ± S.D. Data in (**b**) were analyzed using a repeated measures ANOVA with a Bonferroni multiple comparison post hoc analysis, and data in (**d**) were analyzed with a one-way ANOVA with a Bonferroni post hoc analysis. There were no significant differences in percent weight change among groups; however, footpad swelling was significantly different between the PBS (i.e. PBS-vaccinated and PBS-challenged) control and all other groups except CHIKV-99659 and MAYY/IRES. Additionally, viremia was significantly different between the PBS control and all other groups except CHIKV-99659 and MAYY/IRES on days one and two, and EILV/CHIKV, CHIKV-99659 and MAYY/IRES on days three and four. Statistically significant values are denoted by *p < 0.05.

To investigate the cross-reactive immune potential between CHIKV and MAYV, we first evaluated neutralizing antibody titers in CHIKV-infected C57/B6J mice (Fig. 1a). These mice were vaccinated or inoculated with CHIKV strain 99659, vaccine candidates CHIKV/IRES, EILV/CHIKV, or MAYV/IRES, Venezuelan equine encephalitis virus (VEEV; a distantly related alphavirus) vaccine strain TC-83, the unrelated flavivirus Zika (ZIKV) or were sham-vaccinated (N.B. see materials and methods for details on viruses used). Mice inoculated with CHIKV-99659 and all CHIKV-vaccinated groups developed moderate (>320) CHIKV-neutralizing antibody titers, as expected. Mice sham-vaccinated with PBS served as negative controls and developed no detectable neutralizing antibodies. Mice vaccinated with MAYV/IRES developed high (>640) MAYV-neutralizing antibody titers and mice vaccinated with TC-83 presented with typical VEEV-neutralizing antibody titers (≤320). Mice infected with ZIKV developed low (≤40) ZIKV-neutralizing antibody titers.

Prior to MAYV challenge, little-to-no cross-reactive neutralization activity was detected in the sera of the CHIKV-vaccinated groups, the PBS-vaccinated group, or in the TC-83- and ZIKV-infected groups. Mice infected with wild-type CHIKV-99659 presented the highest (≤160) cross-reactive (MAYV) neutralizing antibody titers out of all non-MAYV vaccine/virus groups, with the CHIKV vaccines generating lower levels of cross-neutralization. Mice were then challenged with 10^5^ PFU of MAYV. There were no significant changes in weight among any of the vaccine/virus groups. This is expected since weight loss offers a less sensitive disease signal in immunocompetent mice as seen previously^41,42^. However, MAYV/IRES completely protected against footpad swelling and CHIKV-99659 also provided significant protection when compared to all other groups (Fig. 1b). These data also correlated with the viremia studies; both MAYV/IRES and CHIKV-99659 vaccination/infection prevented detectable MAYV viremia throughout four days post-challenge (Fig. 1c). There was no evidence for diminished MAYV disease or viremia in any of the CHIKV-vaccinated groups or other virus control groups.

### CHIKV immunity protects against MAYV disease in immunocompromised mice

**Figure 2:**
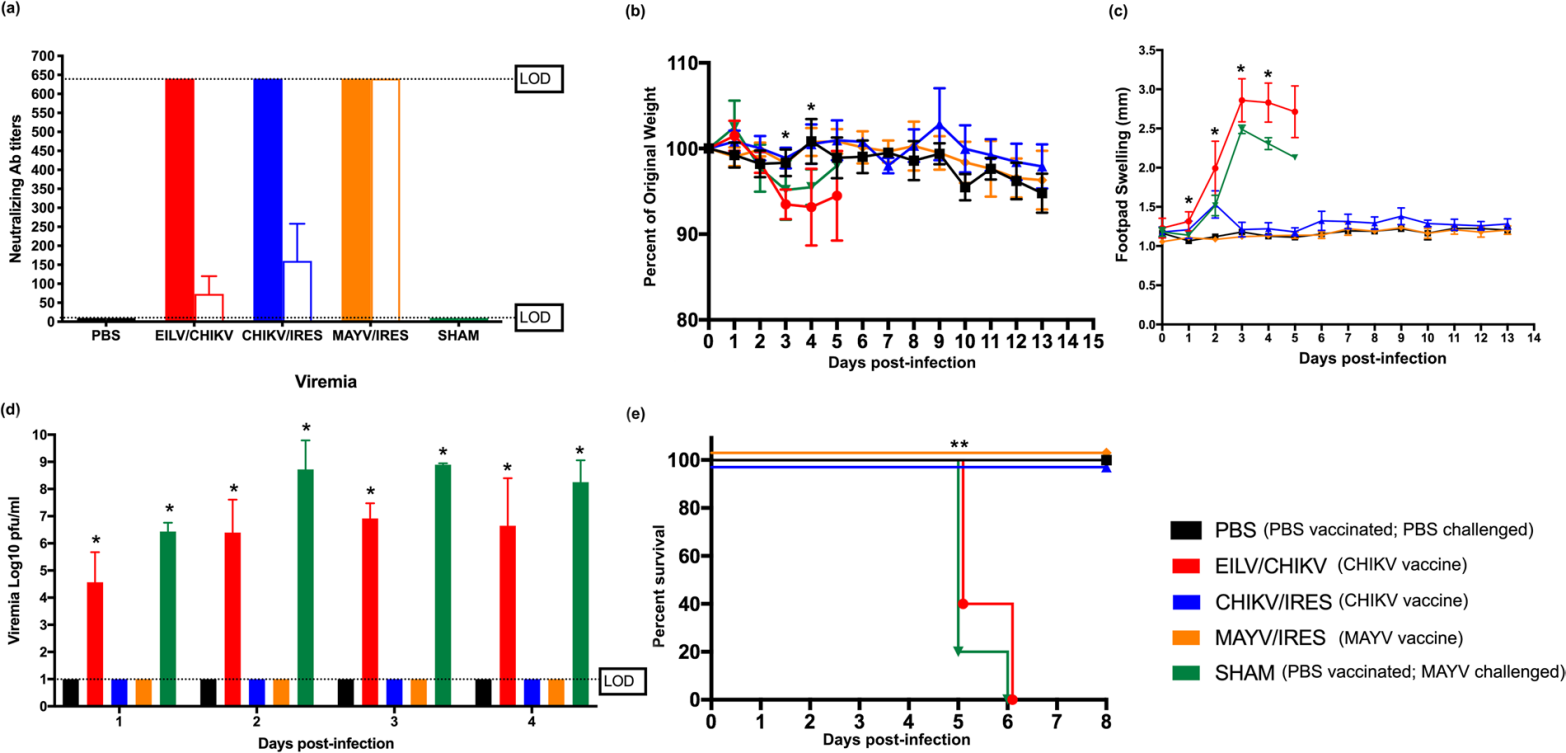
CHIKV immunity provides cross-protection against MAYV infection and disease in an immunocompromised mouse model. (**a**) Virus-specific neutralizing antibody titers (solid bars) and MAYV cross-neutralizing antibody titers (empty bars) were determined prior to MAYV challenge. Dashed line indicates the upper and lower limits of detection (1:640 and 1:20, respectively). (**b**) Weight change, expressed as percent of original, and (**c**) footpad swelling were determined daily throughout the study. (**d**) Viremia was measured daily for four days post-infection and (**e**) survival was recorded. All plotted values in (**b**–**d**) are mean ± S.D. Data in (**b**–**d**) were analyzed using a one-way ANOVA with a Bonferroni post hoc analysis, and survival curves (**e**) were analyzed by Kaplan-Meier survival analysis. Footpad swelling was significantly different between the PBS control (i.e. PBS-vaccinated and PBS-challenged) and EILV/CHIKV groups on days one and two, and between the PBS control and EILV/CHIKV and sham-vaccinated groups on day three, and PBS and sham on day four. Weight change was statistically significant between the PBS control and EILV/CHIKV on days three and four. Viremia and survival were statistically significant for EILV/CHIKV and sham throughout four days post-infection. Statistically significant values are denoted by *p < 0.05.

We next analyzed cross-protective immunity in an immunocompromised, lethal mouse model. A129 mice vaccinated with CHIKV/IRES, MAYV/IRES, EILV/CHIKV, or sham-vaccinated were analyzed for virus-specific neutralizing antibodies. Other virus controls could not be used because of the susceptibility of this model to disease and death following administration of some viruses and vaccines. A129 mice developed high CHIKV neutralization titers (>640) after administration of all CHIKV vaccines, and high MAYV neutralization titers (>640) in the MAYV/IRES group. Interestingly, in A129 mice, we observed similar cross-reactive neutralizing antibody titers (≥80) in the CHIKV/IRES group compared to those of CHIKV-99659 in the immunocompetent mouse model study described above. The EILV/CHIKV group developed less cross-reactive immunity (≤80). Figure 2a describes the virus-specific and MAYV-cross-reactive neutralizing antibody titers for these experimental groups. Following challenge with a lethal dose of MAYV, EILV/CHIKV- and sham-vaccinated groups displayed significant weight loss and footpad swelling (Fig. 2b,c), with mortality resulting five days post-challenge (Fig. 2d). Footpad swelling observations suggested that MAYV/IRES completely protected against disease, as expected, and CHIKV/IRES displayed similar protection with a small spike in footpad swelling on day two post-challenge. Both CHIKV/IRES and MAYV/IRES vaccination protected against detectible MAYV viremia and fatal outcomes. EILV/CHIKV offered no protection from disease (i.e. footpad swelling), viremia, or death.

### Passive transfer of CHIKV immune serum is not protective against MAYV challenge in immunocompromised mice

**Figure 3:**
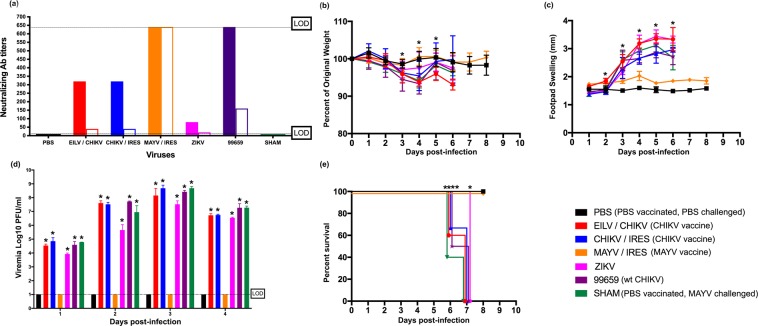
Passive transfer of CHIKV immune sera does not provide cross-protection against MAYV infection and disease in an immunocompromised mouse model. (**a**) Virus-specific neutralizing antibody titers (solid bars) and MAYV cross-neutralizing antibody titers (empty bars) were determined prior to MAYV challenge. Dashed line indicates the upper and lower limits of detection (1:640 and 1:20, respectively). (**b**) Weight change, expressed as percent of original, and (**c**) footpad swelling were determined daily throughout the study. (**d**) Viremia was measured daily for four days post-infection and (**e**) survival was recorded. All plotted values in (**b**–**d**) are mean ± S.D. Data in (b-d) were analyzed using a one-way ANOVA with a Bonferroni post hoc analysis, and survival curves (**e**) were analyzed by Kaplan-Meier survival analysis. When compared to the PBS control (i.e. PBS-vaccinated and PBS-challenged), statistically significant footpad swelling was observed in mice from EILV/CHIKV on days 2–6, CHIKV/IRES on days 1 and 3–6, and ZIKV, CHIKV-99659 and sham on days 3–6. Weight change was statistically significant for EILV/CHIKV on days three and five, and CHIKV-99659 on days three and four, and sham on day four. Viremia (days 1–4) and survival was statistically significant for all groups except MAYV/IRES. Statistically significant values are denoted by * p<0.05.

To further explore whether cross-protective immunity is T-cell- or antibody-mediated, C57/B6J mice were vaccinated or infected with the viruses/vaccines described above in the C57/B6J cross-protection study. Next, 100 µl of immune sera (i.e. collected 6 weeks post-infection) was administered to A129 mice intraperitoneally prior to challenge with a lethal dose (10^5^ PFU) of MAYV. Figure 3a describes the neutralizing antibody titers for the immune sera transferred. No cross-protection was observed among any of the groups except the MAYV/IRES positive control group. Changes in weight were consistent among all groups (Fig. 3b); however, footpad swelling (Fig. 3c) and viremias (Fig. 3d) were elevated in all heterologous CHIKV vaccine/virus groups, and all experimental groups succumbed to disease six days post-MAYV-infection (Fig. 3e). The sham-challenged and MAYV/IRES-vaccinated, challenged control groups survived the entire study and showed no signs of illness.

### T-cells from CHIKV-infected mice are reactive to MAYV 12A antigen

**Figure 4:**
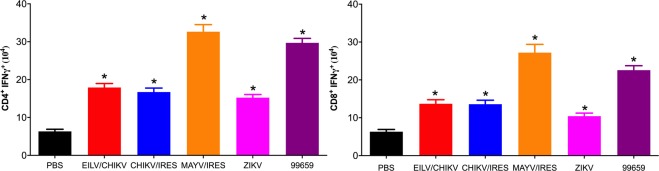
CHIKV immune T-cells are reactive upon stimulation with MAYV 12A. C57/B6J mice were vaccinated or challenged with CHIKV/IRES, EILV/CHIKV, CHIKV-99659, ZIKV, MAYV/IRES or sham-vaccinated with PBS. Six weeks post-vaccination, mice were sacrificed, splenocytes were isolated and stimulated for 24 hours with MAYV, stained with antibodies for CD3, CD4, CD8, IFN-γ and analyzed using flow cytometry. Number of (**a**) CD4^+^ IFN-γ^+^ and (**b**) CD8^+^ IFN-γ^+^ T-cells are plotted as mean ± S.D. Data were analyzed using a one-way ANOVA with a Bonferroni post hoc analysis. Statistically significant values are denoted by *p < 0.05.

To investigate the cellular response to MAYV 12A antigen (i.e. the virus strain used in challenges throughout the study) following exposure to PBS, ZIKV, EILV/CHIKV, CHIKV/IRES, CHIKV-99659, or MAYV/IRES, we measured *ex vivo* intracellular IFN-γ production among murine CD4^+^ and CD8^+^ T-cells following stimulation with MAYV 12A. All groups produced significantly (p ≤ 0.05) higher numbers of MAYV-specific IFN-γ^+^- CD4^+^ and CD8^+^ T-cells when compared to the PBS control group (Fig. 4). As expected, the MAYV/IRES group produced the highest numbers of IFN-γ^+^ T-cells; however, the CHIKV-99659 group also produced large quantities of IFN-γ^+^ T-cells.

### T-cell depletion studies suggest cross-protection is antibody-mediated

**Figure 5:**
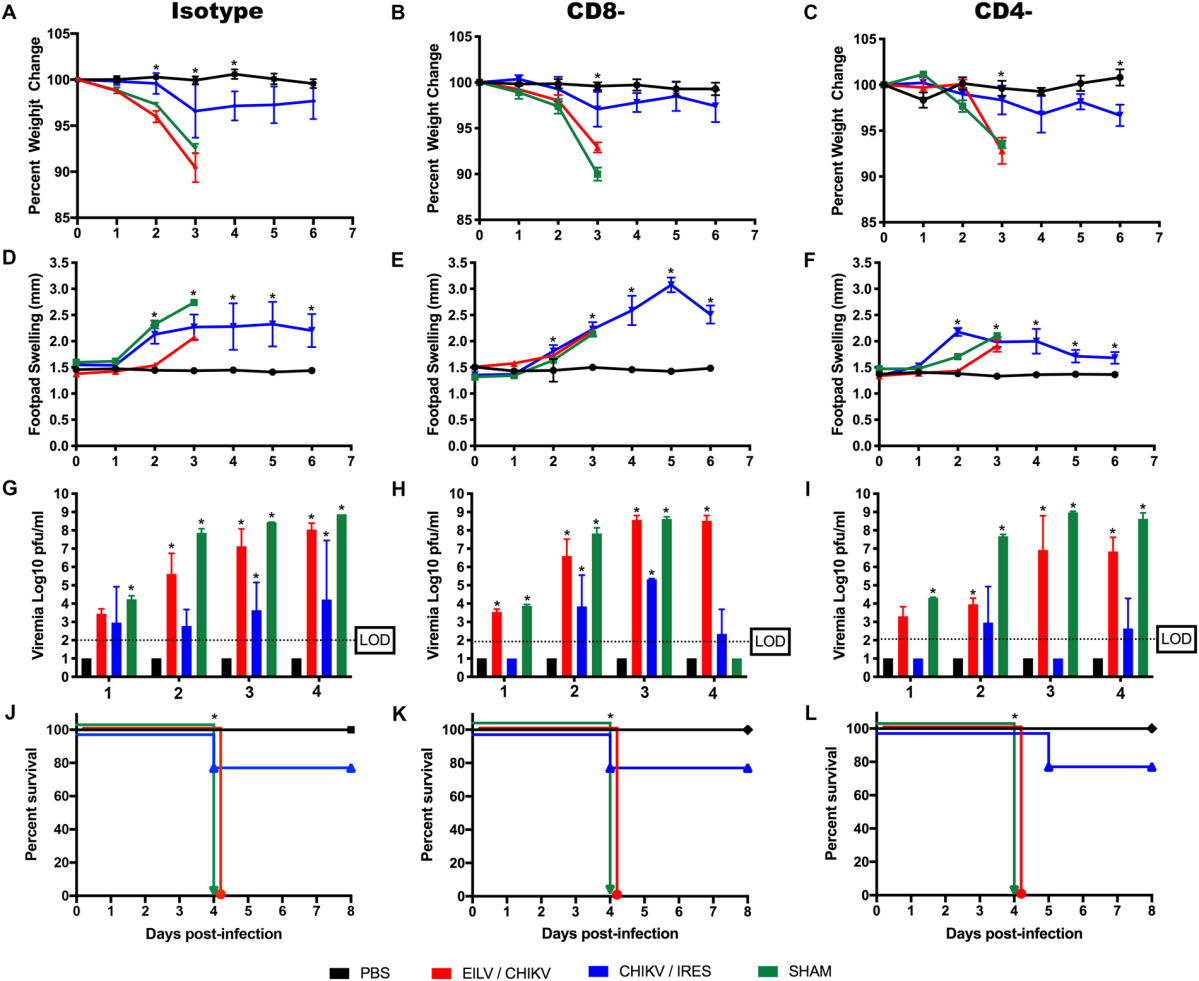
T-cell depletion studies reveal no significant role for CD4^+^ and CD8^+^ T-cells in diminishing MAYV disease. (**a**–**c)** Weight change, expressed as percent of original and (**d**–**f**) footpad swelling were determined daily throughout the study. (**g**–**i**) Viremia was measured daily for four days post-infection, (**j**–**l**) and survival was recorded. All plotted values in (**a**–**i)** are mean ± S.E.M. (**a**–**c**) were analyzed using a repeated measures ANOVA with a Bonferroni multiple comparison post hoc analysis, and data in (**d**–**i**) were analyzed using a one-way ANOVA with a Bonferroni post hoc analysis, and survival curves (**j**–**l**) were analyzed by Kaplan-Meier survival analysis. Statistically significant values are denoted by *p < 0.05.

IFNα/βR^−/−^ mice were vaccinated with 10^4^ PFU of CHIKV/IRES, 10^8^ PFU of EILV/CHIKV or sham-vaccinated with PBS. Ten weeks post vaccination, CD4^+^ and CD8^+^ T-cells were independently depleted with antibodies (>98% depletion efficiency compared to isotype controls; data not shown) and mice were then challenged with a lethal dose (10^5^ PFU) of MAYV. Since previous studies showed that depletion of both CD4^+^ and CD8^+^ T-cells in MAYV/IRES vaccinated A129 mice did not have any effect post MAYV challenge (data not shown), and given the pathogenicity of this vaccine in 6-week old IFNα/βR^−/−^ mice, this group was excluded from this study. As seen previously, EILV/CHIKV vaccination did not protect against MAYV-induced disease, but the CHIKV/IRES group was protected from mortality (Fig. 5). The viremia studies also suggested a reduction in the CHIKV/IRES-vaccinated but not EILV/CHIKV-vaccinated mice (Fig. 5g–i). No statistically significant trend or pattern was detected when CD4^+^ or CD8^+^ depleted groups were compared to the isotype controls. These results suggest little or no role for T cells in the protection against MAYV challenge.

### CHIKV-immune human sera cross-neutralizes MAYV *in-vitro*

To investigate the extent to which naturally acquired CHIKV immunity in humans can cross protect against MAYV infection, 24 serum samples from 20 individuals who presented with suspected CHIKV infection in Trinidad during the 2014/15 CHIKV outbreak (18 individuals) and in 2016 (2 individuals) were screened by ELISA for CHIKV-specific antibodies. These samples were then analyzed for the ability to neutralize CHIKV (i.e. vaccine strain 181/25) and MAYV 12A infection on Vero cells by plaque reduction neutralization tests (PRNT). As indicated in Table 1, both early (*i.e*. <180 days post-onset of illness [dpo]) and late (≥180 dpo) convalescent samples were available for four individuals (TT93, TT103, TT112 and TT114). All other individuals were represented by serum from a single time point, of which four were during early convalescence (TT35, TT47, TT107, and TT122). CHIKV-specific IgG antibodies were detected in 22 out of the 24 samples, and CHIKV-specific IgM antibodies in one out of ten samples tested (i.e. TT47). CHIKV-neutralizing antibodies were detected in all of the CHIKV IgG-positive samples with PRNT_80_ titers ranging from 40 to 40,960. All but two of the CHIKV IgG-positive sera (i.e. TT103 and TT112) also neutralized MAYV with titers ranging from 40 to 2,560. Two samples collected during the CHIKV outbreak (TT10 and TT38) had eight- and two-fold higher PRNT_80_ titers for MAYV 12A than for CHIKV 181/25 respectively, suggesting possible MAYV or another alphavirus infection.

**Table 1:** Serological status and PRNT_80_ titers of human sera from individuals with suspected CHIKV infections.

Sample ID	Days post onset of illness (dpo)	CHIKV serostatus		PRNT_80_ titer	
		IgG	IgM	CHIKV	MAYV
TT10	312	Positive	—	**40**	**320**^**#**^
TT13	336	Negative	—	<20	<20
TT35	135*	Positive	—	**2560**	**2560**
TT38	210	Positive	—	**320**	**640**
TT39	210	Positive	—	**2560**	**640**
TT47	130*	Positive	Positive	**5120**	**640**
TT48	330	Positive	—	**10240**	**640**
TT49	300	Positive	—	**10240**	**640**
TT52	360	Positive	—	**10240**	**640**
TT54	330	Positive^†^	—	**2560**	**640**
TT57	360	Positive	—	**40960**	**640**
TT65	363	Positive	—	**640**	**640**
TT68	389	Positive	—	**640**	**160**
TT92	360	Positive	—	**640**	**320**
TT93	30*	Negative	Negative	**40**	<20
	367	Negative	—	<20	<20
TT103^§^	30*	Positive	Negative	**640**	<20
	360	Positive	—	**320**	**40**
TT107^§^	30*	Positive	Negative	**640**	**40**
TT112	20*	Positive	NT	**80**	<20
	427	Positive	—	**640**	**80**
TT114	10*	Positive	Negative	**640**	**160**
	415	Positive	—	**640**	**160**
TT122	23*	Positive	Negative	**640**	**40**

*Indicates early convalescent samples (i.e. serum samples collected less than 180 dpo). NT indicates “not tested”, “ – “ indicates “not applicable”. ^†^Results of IgG ELISAs were discordant (positive by AbCam and negative by EuroImmun Anti-Chikungunya Virus ELISA). ^§^Presented with suspected CHIKV in 2016; all others presented during 2014/15 CHIKV epidemic period. ^#^Indicates a possible MAYV or another alphavirus infection.

## Discussion

MAYV is an important re-emerging alphavirus in the Americas. Despite its history of small sporadic outbreaks, MAYV has the potential to cause large devastating outbreaks through potential peridomestic, human-amplified transmission. Therefore, identifying and understanding the factors that can influence the epidemic potential of MAYV is important to forecasting its spread and disease burden. MAYV and CHIKV share many characteristics and are phylogenetically and serologically related members of the Semliki Forest complex. There is no published information available on whether or not MAYV circulation or immunity is affecting the epidemic dynamics of CHIKV in the Americas, but CHIKV is now probably endemic in many parts of Latin America and its reciprocal effects on MAYV emergence warrants investigation.

Our studies in immunocompetent C57/B6J mice show that immunity derived from wild-type CHIKV (CHIKV-99659) infection diminished MAYV disease and completely prevented MAYV viremia. However, immunity induced by two highly immunogenic and efficacious CHIKV vaccines, CHIKV/IRES or EILV/CHIKV, offered no protection from MAYV disease or viremia. The differential protective effects observed here may be attributed to the significant disparity in CHIKV-specific and MAYV cross-reactive neutralizing antibody titers. The sera from CHIKV-99659-infected mice contained CHIKV-neutralizing antibody titers >640, at least double those of the vaccinated groups. As expected, the degree of CHIKV immunity significantly correlates with MAYV cross-reactive neutralizing antibody titers, such that higher CHIKV-specific neutralizing antibody titers are correlated with higher cross-reactive neutralizing antibody titers. Our data also suggest a cross-neutralizing antibody threshold needed for protection from MAYV infection. The complete absence of any protective effects by the TC-83 groups, which are either more distantly related or unrelated viruses, respectively, confirms that the observed CHIKV cross-protection is specific and likely due to antigenic overlap between CHIKV and MAYV. Immunity to CHIKV-99659 infection also completely prevented the development of MAYV viremia, which has important implications for MAYV’s emergence potential since the absence of or even reduction of MAYV viremia in CHIKV-immune persons could prevent transmission. This suggests that CHIKV herd immunity, which is high in many parts of Asia^43–45^ and Latin America^46–48^ may reduce MAYV’s epidemic potential.

Our cross-protection study performed in 8-wk old A129 mice, a more sensitive model for MAYV disease, also demonstrated limited cross-protection from CHIKV vaccines. Of the vertebrate replication-competent viruses, only the attenuated CHIKV/IRES and MAYV/IRES vaccine strains could be employed because of the high susceptibility of this model to fatal disease with more virulent virus strains. The vertebrate replication-incompetent EILV/CHIKV-derived immunity provided no protection against MAYV viremia or mortality, but in fact exacerbated weight loss and footpad swelling in this model. Although sub-neutralizing levels of cross-reactive antibodies against MAYV could result in antibody dependent enhancement (ADE) of disease as experimentally demonstrated with CHIKV^49^ and Ross River virus^50–52^, there is no evidence of this phenomenon for alphavirus infection of humans. Further experimental and epidemiological research is required to explore the possibility of ADE among antigenically related alphavirus infections.

In contrast, CHIKV/IRES-derived immunity protected against MAYV viremia and mortality, and greatly reduced footpad swelling on day two post-challenge. Remarkably, evaluation of CHIKV-specific and MAYV-cross-reactive neutralizing antibody titers from the CHIKV/IRES group closely reflected those induced by CHIKV-99659 infection of C57/B6J mice. The enhanced susceptibility of this immunocompromised model to viral replication in the absence of an interferon type I response presumably allows a live-attenuated vaccine such as CHIKV/IRES to illicit immunity similar to that following a wild-type CHIKV infection in immunocompetent mice, and hence a similar degree of cross-protection was observed. Our study suggests that a minimum CHIKV immunity threshold must be achieved to provide effective cross-protection against MAYV disease. Our data suggests that cross-protection may be conferred when a cross-neutralization threshold of ≥80 is achieved *in vivo*.

Given the evidence that CHIKV immunity can reduce MAYV disease and prevent MAYV viremia, thereby hindering its emergence potential, we sought to determine the influence of humoral and cell-mediated immunity on the observed cross-protection. There were high numbers of MAYV antigen-cross reactive T-cells in CHIKV/IRES-vaccinated and wild-type CHIKV-infected mice suggesting shared T-cell epitopes among the viruses. With the exception of MAYV/IRES, passive transfer of immune sera from EILV/CHIKV-, CHIKV/IRES-, CHIKV-99659-, TC-83-, and ZIKV-infected mice provided no protection from MAYV disease in A129 mice. Given IACUC protocol limitations, circulating neutralizing antibody titers were not estimated after passive transfer, however, previous studies indicate that transferring a neutralizing antibody titer of 320 or 640, typically results in a circulating titer of 20–40 or 40–80. This result can therefore be explained by the considerable dilution of the neutralizing antibodies circulating within the mouse (≈10-fold dilution), such that the minimum antibody threshold needed for protection was not obtained. To ensure that the previously observed minimum neutralizing antibody threshold was maintained, T-cell depletion studies were performed in IFNα/βR^−/−^mice. As expected, these studies generated similar results with the initial cross-protection studies done in the 8-week A129 mice with an intact T-cell response, although this cellular response was possibly primed to a lesser extent given the longer period (4 weeks versus 10 weeks) between CHIKV exposure and MAYV challenge. Consistent with our previous studies, EILV/CHIKV vaccination failed to protect against footpad swelling, weight loss, or viremia, while, CHIKV/IRES-vaccinated mice depleted of T-cells survived challenge and exhibited reduced viremia. The absence of any significant trend in the knockout models indicated that CD4^+^ and/or CD8^+^ T-cells have little or no effect on the immune protection observed. These data suggest that neutralizing antibodies against CHIKV are sufficient to reduce MAYV disease and prevent viremia. Although many studies demonstrate that humoral immunity alone is adequate to provide homologous and heterologous protection from alphaviral disease^34–36,53,54^, previous work provides strong evidence of the role of T-cells in homologous and heterologous protection among members of the Semliki Forest complex^34,38,40,55^. While our mechanistic studies suggest the humoral immune response alone can provide protection and T-cells play little or no role in diminishing disease, further studies such as adoptive transfers may be warranted to determine the exact degree, if any, of cross-protection afforded specifically by the cellular immune response.

Finally, our results indicate that naturally acquired CHIKV-specific human antibodies from Trinidad can strongly cross-neutralize MAYV infection *in vitro*. Although our sample size was small, a recent report based on sera from 35 individuals also supports our findings^56^. Our neutralization titers were generally higher (at least four-fold) against CHIKV than MAYV, delineating CHIKV infections in these individuals. Of note, there were two individuals whose neutralization titers were eight- and two-fold higher against MAYV than CHIKV indicating possible MAYV or another alphaviral infection respectively. No MAYV outbreaks have been documented in Trinidad, but the virus was originally isolated in forested regions within Trinidad^1^ and presumably remains in circulation. Typical MAYV cross-neutralization titers in humans were above the apparent PRNT_80_ threshold of 80 for cross-protection in mice (Table 1), suggesting that cross-protection may be conferred in human samples. However, further studies aimed at elucidating the cross-protection PRNT titer threshold of human serum are warranted to test this hypothesis. There is currently insufficient epidemiologic data available throughout the Americas to determine with any certainty whether MAYV emergence has been limited by preexisting CHIKV immunity since 2013. This is further compounded by the absence of adequate diagnostics for MAYV or inclusion of MAYV as a differential diagnostic in some countries. Improved epidemiological studies with rigorous and thorough diagnostic testing are necessary to provide sufficient information to further test this hypothesis.

In conclusion, our results indicate a significant cross-reactive immunological response between MAYV and CHIKV, which could reduce the risk of MAYV emergence in neotropical regions with high levels of natural CHIKV herd immunity due to epidemic transmission since 2013. However, cross-reactivity based on a CHIKV vaccine may not be strong enough to limit the emergence of MAYV. MAYV-specific vaccines should therefore continue to be developed.

## Materials and Methods

### Viruses and cell cultures

The wild-type and attenuated vaccine CHIKV strains used in this study were provided by the World Reference Center for Emerging Viruses and Arboviruses (WRCEVA) at the University of Texas Medical Branch (Galveston, Texas, USA). These included CHIKV/IRES (i.e. a live-attenuated CHIKV vaccine)^57^, EILV/CHIKV (i.e. a chimeric host-restricted CHIKV vaccine)^58^, CHIKV-99659 (i.e. an Asian-American, wild-type CHIKV isolated from the British Virgin Islands in 2014)^59^ and CHIKV 181/25 (i.e. vaccine strain)^60^. MAYV strains included MAYV/IRES (i.e. a live-attenuated MAYV vaccine)^41^ and 12A (i.e. a genotype D wild-type MAYV isolate from a 2010 outbreak in Venezuela)^10^. Additionally, Zika virus (ZIKV) strain FSS13025 (i.e. a Cambodian ZIKV strain)^61^ and Venezuelan equine encephalitis virus (VEEV) strain TC-83 (i.e. a live-attenuated VEEV vaccine)^62,63^ were used for the study. Vero 76 (African green monkey kidney), were obtained from the American Type Cell Collection (Bethesda, MD) and maintained in Dulbecco’s minimal essential medium (DMEM) supplemented with 5% fetal bovine serum (FBS), and Penicillin and Streptomycin (Pen/Strep) (100 U/ml) in a 37°C, 5% CO_2_ incubator. C7/10 (*Aedes Albopictus*) cells were obtained from the WRCEVA and maintained in DMEM supplemented with 10% FBS, 1% minimal essential medium non-essential amino acids, 1% tryptose phosphate broth and Pen/Strep (100 U/ml) in a 29°C, 5% CO_2_ incubator. C7/10 cells were used to propagate EILV/CHIKV (i.e. it does not replicate in vertebrate cells) for vaccination and Vero cells were used to propagate all remaining viruses and vaccine strains, and used during plaque assays and PRNT_80_ tests.

### Cross protection in an immunocompetent mouse model

Four-week old C57B6/J mice (Jackson Laboratory, Bar Harbor, ME, n = 8) were vaccinated or infected subcutaneously with 10^4^ plaque-forming units (PFU) of CHIKV/IRES, CHIKV-99659, MAYV/IRES, TC-83, 10^5^ PFU of ZIKV, 10^8^ PFU of EILV/CHIKV, or sham-vaccinated with PBS. A PBS group (n = 8) was also employed as uninfected controls. Virus and vaccine doses were administered based on their initial reports to achieve sufficient preexisting immunity for homologous protection. Twenty-eight days post-vaccination, mice were bled to evaluate humoral immune responses via neutralization assays. Thirty-one days post-vaccination mice were challenged intradermally in the hind footpad with 10^5^ PFU of MAYV. This dose was selected based on previous studies to generate sufficient MAYV disease signal to allow for statistical comparison in both murine models employed^41,42^. Mice were bled on alternating days (i.e. days one and three or days two and four) for viremia measures. Viremia was determined by plaque assay using Vero cells with standard protocols and eighty percent plaque reduction neutralization tests (PRNT_80_) were also performed on Vero cells using CHIKV-99659 as the control CHIKV and 12A as the control MAYV^64^. For PRNT_80_, serum was heat inactivated at 56°C for 30 min and serially diluted in DMEM containing 1% FBS. All dilutions were then further diluted an additional 2-fold by the addition of ∼100 PFU of MAYV or CHIKV, mixed, and incubated at 37°C for 1 hr, then 100 PFU was transferred to 90% confluent monolayers of Vero cells in 12-well plates and incubated at 37°C for 1 hr. PRNTs were done in duplicate. Overlay containing 0.4% agarose in DMEM with 2% FBS and Penicillin and Streptomycin (Pen/Strep) (100 U/ml) was added, and plates were incubated for 2 days at 37 °C. Cells were then fixed in 10% formaldehyde, and plaques visualized following crystal violet staining. Weight changes and footpad swelling were measured daily for 14 days post-challenge.

### Cross protection in an immunocompromised mouse model

A129 (i.e. Interferon alpha/beta receptor null) mice were bred locally at UTMB. At four weeks of age, mice (n = 5 or 6) were vaccinated with 10^4^ PFU of CHIKV/IRES, MAYV/IRES, 10^8^ PFU of EILV/CHIKV, or sham-vaccinated with PBS. A PBS group (n = 5) was also employed as uninfected controls. Twenty-eight days post-vaccination, mice were bled for neutralization assays, and then challenged as described above 31 days post-vaccination with a lethal dose (10^5^ PFU) of MAYV. Mice were bled for viremia as described above, and weights and footpad swelling were measured until mortality.

### Passive transfers of immune sera to an immunocompromised mouse model

At four weeks of age, C57/B6J mice (n = 4) were vaccinated or infected with 10^4^ PFU of CHIKV/IRES, CHIKV-99659, MAYV/IRES, 10^5^ PFU of ZIKV, 10^8^ PFU of EILV/CHIKV or sham-vaccinated with PBS. A PBS group (n = 5) was also employed as uninfected controls. Six weeks post-vaccination, mice were terminally bled to measure neutralizing antibody titers and to collect immune sera for passive transfers. Sera from each group were pooled and PRNT_80_ were completed using respective antigens. 100 µl of pooled sera were administered intraperitoneally to A129 mice (n = 5 or 6) 36 hours prior to challenge with a lethal dose (10^5^ PFU) of MAYV. Mice were bled daily for four days for viremia measurements. Weight changes and footpad swelling were measured for 14 days post-challenge or until mortality.

### T-cell stimulation assays

C57BL/6J mice were vaccinated or infected with CHIKV/IRES, EILV/CHIKV, CHIKV-99659, ZIKV, MAYV/IRES, or sham-vaccinated with PBS using doses described above. Six weeks post-vaccination, mice were sacrificed and spleens were collected for splenocyte isolation. Splenocytes were stimulated for 24 hours with MAYV 12A (MOI = 1) in a Golgi-plug (BD Biosciences, San Jose, CA) containing medium. Cells were then harvested, stained with antibodies for CD3, CD4, or CD8, fixed, and permeabilized with BD Cytofix/Cytoperm (BD Biosciences, San Jose, CA) before adding PE-conjugated anti-IFN-γ, or control PE-conjugated rat IgG1 (eBiosciences, San Diego, CA). Cells were then washed and analyzed using a C6 Flow Cytometer (Accuri cytometers, Ann Arbor, MI).

### T-cell depletion studies

Five-week-old IFNAR knockout mice (n = 15) were vaccinated with 10^4^ PFU of CHIKV/IRES, 10^8^ PFU of EILV/CHIKV, or sham-vaccinated with PBS. Ten weeks later, anti-CD4 (clone GK1.5), anti-CD8 (clone 2.43), or rat IgG2b isotype control (clone LTF-2) antibodies (Bio X Cell, West Lebanon, NH) were administered intraperitoneally to mice (e.g. n = 5/antibody group) on days −3, −1, and 3 post-challenge. On the day of challenge, blood samples were taken from three mice per each group and circulating T-cells were quantified by flow cytometry to determine knockdown efficiency. After confirming >95% knockdown in all groups, mice were challenged with a lethal dose (10^5^ PFU) of MAYV. Mice were bled daily for four days for viremia measurements. Weight changes and footpad swelling were measured for six days post-challenge or until mortality.

### Cross-neutralization studies of MAYV 12A in human sera samples

Twenty-four (24) serum samples from 20 individuals who had presented with clinical symptoms of CHIKV infection during the 2014–2015 CHIKV epidemic in Trinidad (n = 18) and in 2016 (n = 2) were screened for neutralizing activity against CHIKV vaccine strain 181/25 and MAYV strain 12A using PRNTs. Neutralization titers were calculated and expressed as the reciprocal of the initial serum dilution yielding greater than 80% reduction (PRNT_80_) in the number of plaques as compared to control wells. Serum samples were tested for the presence of CHIKV specific antibodies using EuroImmun Anti-Chikungunya Virus ELISA IgM and IgG kits (EuroImmun AG, Germany), with the IgM ELISA used only for early convalescent (<180 dpo) samples. A subset of samples (TT10 – TT57) were also tested using AbCam Human Anti-Chikungunya Virus ELISA kits (AbCam, Cambridge, UK) for IgG. Samples were deemed IgG positive if a positive result was returned by either the EuroImmun ® or AbCam ® IgG kit.

### Statistics

Data normalcy was tested using a combination of Q-Q plot and box-plot analyses. However, uneven group numbers, especially in the context of lethal models, precluded ranks-based analyses and parametric tests were universally employed. One-way and repeated measures ANOVAs, as well as Kaplan-Meier survival curves were performed using SPSS statistics software (IBM Corporation, Armonk, NY).

### Ethical approval and informed consent

All experimental protocols were approved by the Virginia Tech Institutional Biosafety Committee. All animal study protocols and experiments were approved by Virginia Tech’s Institutional Animal Care and Use Committee (IACUC). All animal experiments were performed in compliance with the guidelines of the Virginia Tech’s IACUC. The study protocols regarding archived human sera samples were approved by the Ethics Committee of the University of the West Indies, St. Augustine campus, and methods use were in accordance with the relevant guidelines and regulations. Written informed consent was obtained from all participants. Written informed consent was obtained from all participants.

## Data Availability

All reagents, data and associated protocols are available to readers upon request.
